# Variations of the quality of care during the COVID-19 pandemic affected the mortality rate of non-COVID-19 patients with hip fracture

**DOI:** 10.1371/journal.pone.0263944

**Published:** 2022-02-16

**Authors:** Davide Golinelli, Francesco Sanmarchi, Angelo Capodici, Giorgia Gribaudo, Mattia Altini, Simona Rosa, Francesco Esposito, Maria Pia Fantini, Jacopo Lenzi

**Affiliations:** 1 Department of Biomedical and Neuromotor Sciences (DIBINEM), Alma Mater Studiorum–University of Bologna, Bologna, Italy; 2 Healthcare Administration, AUSL Romagna, Ravenna, Italy; University Hospital Zurich, SWITZERLAND

## Abstract

**Introduction:**

As COVID-19 roared through the world, governments worldwide enforced containment measures that affected various treatment pathways, including those for hip fractures (HFs). This study aimed to measure process and outcome indicators related to the quality of care provided to non-COVID-19 elderly patients affected by HF in Emilia-Romagna, a region of Italy severely hit by the pandemic.

**Methods:**

We collected the hospital discharge records of all patients admitted to the hospitals of Emilia-Romagna with a diagnosis of HF from January to May in the years 2019 (pre-pandemic period) and 2020 (pandemic period). We analyzed surgery rate, surgery delays, length of hospital stay, timely rehabilitation, and 30-day mortality for each HF patient. We evaluated monthly data (2020 vs. 2019) with the chi-square and t-test, where appropriate. Logistic regression was used to investigate the differences in 30-day mortality.

**Results:**

Our study included 5379 patients with HF. In April and May 2020, there was a significant increase in the proportion of HF patients that did not undergo timely surgery. In March 2020, we found a significant increase in mortality (OR = 2.22). Male sex (OR = 1.92), age ≥90 years (OR = 4.33), surgery after 48 hours (OR = 3.08) and not receiving surgery (OR = 6.19) were significantly associated with increased mortality. After adjusting for the aforementioned factors, patients hospitalized in March 2020 still suffered higher mortality (OR = 2.21).

**Conclusions:**

There was a reduction in the overall quality of care provided to non-COVID-19 elderly patients affected by HF, whose mortality increased in March 2020. Patients’ characteristics and variations in processes of care partially explained this increase. Policymakers and professionals involved in the management of COVID-19 patients should be aware of the needs of patients with other health needs, which should be carefully investigated and included in future emergency preparedness and response plans.

## Introduction

Timely detection, intervention and rehabilitation are key factors for the successful management of patients who suffer from hip fractures (HFs) [1–4].To improve functional outcomes and reduce mortality, clinical guidelines recommend performing surgical repair or replacement of HF within 48 hours of hospital admission, or as soon as the patient is medically stable, avoiding a delay in surgery, ensuring early mobilization, and providing a post-acute rehabilitation plan after a few days of hospital stay [5–7].

In 2020, the COVID-19 pandemic forced governments worldwide to enforce containment measures such as social distancing and home quarantining. These measures had a considerable impact on various treatment pathways [8, 9], including those for HF [10]. During the initial phases of the pandemic, elective surgery was stopped in many healthcare systems, and only emergencies were treated [11]. In many hospitals, wards were merged, and non-COVID-19 cohorts were created to reduce cross-infection between staff members and patients [12]. Early discharge of HF patients was encouraged to increase the number of available beds, reallocate clinical staff, and ensure patient and staff members’ safety standards.

As COVID-19 roared across the world, treatment pathways for HF felt the blow and musculoskeletal facilities were reorganized as a result, patients were at risk of being left without proper care and were exposed to increased disability and mortality [13–15]. Some healthcare systems withstood the impact, maintaining levels of care for non-COVID-19 patients similar to those of the pre-pandemic period. Other systems, such as the Italian one, were caught unprepared [16] and this affected their performance at both the hospital and out-of-hospital level [17].

After the first case of COVID-19 on February 21, 2020, the first wave of the pandemic struck all over northern Italy, including Emilia-Romagna (see S1 Fig). Italy’s national government decided to promptly implement strict non-pharmaceutical measures. National quarantine was declared, and the entire country was under lockdown from March 9 to May 4; complete freedom of movement was not reintroduced until June 3, 2020 [12]. These restrictive measures affected schools, universities, bars, and restaurants, and determined the disruption of usual healthcare pathways [17], as healthcare systems had to react to the emergency, suspending non-urgent surgical interventions, outpatient visits, and many primary services.

Healthcare systems’ ability to adapt to the mutating population’s health needs caused by an emergency such as the COVID-19 pandemic can be assessed through several health processes and outcome indicators [18]. Accordingly, it is vital to monitor healthcare systems’ resilience, particularly during periods of high distress, and to investigate whether such indicators can be useful to evaluate healthcare systems’ capacity and describe systems’ resilience during emergencies.

Given the forced reorganization of healthcare services during the first wave of the pandemic, we aimed to measure process and outcome indicators related to the quality of care provided to non-COVID-19 elderly patients affected by HFs in Emilia-Romagna. Specifically, we analyzed the length of hospital stay (LOS) as well as the rates of and delays in surgical treatment/rehabilitation as process indicators, and 30-day mortality as the main health outcome.

## Materials and methods

### Setting of the study

The Italian national health service (Servizio Sanitario Nazionale) is a universalistic health system funded through general taxation. Emilia-Romagna is one of the largest regions of northern Italy, with ~4.5 million inhabitants as of 2020. Its regional health system includes 8 local health trusts, 4 university hospitals, 1 general hospital trust, and 4 research hospitals.

In 2013, Emilia-Romagna improved the management of patients with HF, reducing delays in surgery and designing specific modalities for post-operative rehabilitation [19].

### Study design and population

In this retrospective cohort study, we gathered the hospital discharge records (HDRs) of all patients aged ≥65 admitted to the hospitals of Emilia-Romagna with a primary or secondary diagnosis of HF (ICD-9-CM code 820.xx) between January and May 2020 (study period) and between January and May 2019 (control period). In Italy, 2007 ICD-9-CM is the official system of assigning codes to diagnoses and procedures associated with hospital utilization, and hospital cases are classified into 538 homogeneous and mutually exclusive groups using the Medicare diagnosis-related group (DRG) system, version 24.0.

We excluded non-residents in Emilia-Romagna, transfers from other hospitals, polytraumas (DRG 484–487), diagnoses or history of malignant tumors (ICD-9-CM code 140.0–208.9x, 238.6, V10.xx), and cases of COVID-19 using the criteria issued on March 10, 2020, by Italy’s Ministry of Health (ICD-9-CM code V01.82, 079.82, 480.3, V07.0). We excluded patients who died within 1 day of hospital admission without surgery, and those directly admitted to spinal injury units, rehabilitation hospitals or long-term care facilities.

We collected from the health administrative databases of Emilia-Romagna the drug prescriptions of each patient over a lookback period of 3 years to compute the Modified Chronic Disease Score (M-CDS), a drug-based index that has been shown to be a good predictor of 1-year mortality [20].

### Processes of care

Process indicators included LOS, HF surgery (within 2 days, after 2 days or never performed), and rehabilitation within 30 days of hospital admission, i.e., facility bed-based rehabilitation programs after HF provided in public or private hospitals, intermediate care facilities, community hospitals or nursing homes.

Hip-fracture surgery was defined as any of the following procedures registered in the HDRs: closed reduction of fracture without internal fixation (ICD-9-CM codes 79.00, 79.05); closed reduction of fracture with internal fixation (79.10, 79.15); open reduction of fracture without internal fixation (79.20, 79.25); open reduction of fracture with internal fixation (79.30, 79.35); total or partial hip replacement (81.51, 81.52).

Through data linkage, we were able to track whether post-acute patients entered bed-based rehabilitation programs in public or private hospital units, community hospitals, or nursing home beds in residential care facilities for the elderly.

### Outcome

The health outcome under study was 30-day mortality, i.e., all-cause death within 30 days of hospital admission, either inside or outside the hospital.

### Statistical analysis

Numerical variables were summarized as mean ± standard deviation; categorical variables were summarized as counts (percentages). Comparisons of patient characteristics and process indicators between 2020 and 2019 were investigated with the chi-squared or *t*-test. Differences in 30-day mortality were expressed as odds ratios (ORs) with 95% confidence intervals (CIs), and were adjusted by age, sex and M-CDS via multivariable logistic regression analysis.

All analyses were stratified by month of the year. If a significant difference was present between 2020 and 2019, we further adjusted the analysis by including HF surgery and LOS as additional covariates in the multivariable logistic regression model.

All analyses were performed using SPSS 26.0 (Armonk, NY: IBM Corp) and Stata 15 (College Station, TX: StataCorp LLC). The significance level was set at 0.05.

### Ethical approval

This study was approved by the *Comitato Etico Indipendente di Area Vasta Emilia Centro* (approval: April 17, 2019; amendment: March 22, 2021). This retrospective study was carried out in conformity with the regulations on data management with the Italian law on privacy (Legislation Decree 196/2003 amended by Legislation Decree 101/2018). Data were pseudonymized prior to the analysis and each patient was assigned a unique identifier that does not allow to trace the patient’s identity or other sensitive data. Pseudonymized administrative data can be used without a specific written informed consent when patient information is collected for healthcare management and healthcare quality evaluation and improvement (according to art. 110 on medical and biomedical and epidemiological research, Legislation Decree 101/2018). All procedures performed in this study were in accordance with the 1964 Helsinki Declaration and its later amendments. Data used in this research were obtained from the Regional Healthcare Information System, which includes detailed information on the use of healthcare services by all regional patients. The study, based on routine administrative information, was carried out in conformity with the regulations on data management of Emilia-Romagna and with Italian privacy law.

## Results

Table 1 shows the characteristics of the 5379 patients with HF included in the study (2531 [47.1%] in Jan-May 2020 and 2848 [52.9%] in Jan-May 2019). Most patients were female (74.2%) and mean age was 84.3 ± 7.4 years. No significant differences were found in the distribution of age, sex and M-CDS between 2019 and 2020. However, we observed a significant decrease in hospital admissions for HF in March and April 2020 as compared with the same months of the previous year (–18.9% [430 vs. 530] in March and –25.6% [445 vs. 598] in April).

**Table 1:** Demographic and clinical characteristics of the study population, by year and month of the year, Emilia-Romagna, Italy.

	Year of admission		*P*-value*
	2020	2019	
January			
Hip fractures	583	621	0.273
Age, y	84.3 ± 7.6	84.5 ± 7.2	0.553
Female	430 (73.8)	451 (72.6)	0.658
M-CDS	6.3 ± 5.0	5.9 ± 5.1	0.173
February			
Hip fractures	522	508	0.663
Age, y	83.8 ± 7.2	84.4 ± 7.4	0.244
Female	385 (73.8)	375 (73.8)	0.981
M-CDS	6.4 ± 5.1	6.2 ± 5.0	0.520
March			
Hip fractures	430	530	0.001
Age, y	84.7 ± 7.3	84.6 ± 7.5	0.726
Female	320 (74.4)	412 (77.7)	0.230
M-CDS	6.5 ± 5.0	5.9 ± 4.9	0.064
April			
Hip fractures	445	598	<0.001
Age, y	84.3 ± 7.4	83.8 ± 7.3	0.285
Female	335 (75.3)	430 (71.9)	0.223
M-CDS	6.1 ± 5.3	6.3 ± 5.3	0.674
May			
Hip fractures	551	591	0.237
Age, y	83.8 ± 7.5	84.1 ± 7.4	0.551
Female	403 (73.1)	449 (76.0)	0.272
M-CDS	6.0 ± 5.1	6.1 ± 5.1	0.628
Jan-May			
Hip fractures	2531	2848	<0.001
Age, y	84.2 ± 7.4	84.3 ± 7.4	0.633
Female	1873 (74.0)	2117 (74.3)	0.782
M-CDS	6.2 ± 5.1	6.1 ± 5.1	0.211

* Obtained with chi-squared test or t-test, where appropriate.

Note: Values are count (percentage) or mean ± standard deviation.

M-CDS, Modified Chronic Disease Score.

As shown in Table 2, in April and May 2020 there was a significant increase in the proportion of HFs not treated with surgery, as compared with April (+7.1% [10.6% vs. 3.5%]) and May 2019 (+4.5% [8.9% vs. 4.4%]), coupled with a significant reduction in the proportion of operations performed within 2 days (–5.5% [70.1% vs. 75.6%] in April and –7.5% [67.3% vs. 74.8%] in May). Mean LOS was significantly lower in March, April and May 2020 as compared with the same months of the previous year (–1.8 days [9.9 vs. 11.7] in March, –1.7 days [10.9 vs. 12.6] in April, and –1.0 days [11.3 vs. 12.3] in May).

**Table 2:** Hip-fracture surgery, length of stay, and rehabilitation by year and month of the year, Emilia-Romagna, Italy.

	Year of admission		*P*-value*
	2020	2019	
January			
Surgery within 2 days	465 (79.8)	487 (78.4)	0.823
Surgery after 2 days	94 (16.1)	105 (16.9)	·
No surgery	24 (4.1)	29 (4.7)	·
Length of stay, d	12.4 ± 6.4	12.4 ± 8.0	0.952
Rehabilitation within 30 days	355 (62.5)	381 (63.6)	0.370
February			
Surgery within 2 days	437 (83.7)	411 (80.9)	0.245
Surgery after 2 days	62 (11.9)	78 (15.4)	·
No surgery	23 (4.4)	19 (3.7)	·
Length of stay, d	12.4 ± 6.9	12.4 ± 7.1	0.842
Rehabilitation within 30 days	293 (57.9)	315 (64.3)	0.023
March			
Surgery within 2 days	344 (80.0)	407 (76.8)	0.479
Surgery after 2 days	64 (14.9)	90 (17.0)	·
No surgery	22 (5.1)	33 (6.2)	·
Length of stay, d	9.9 ± 7.0	11.7 ± 8.7	<0.001
Rehabilitation within 30 days	178 (43.2)	331 (64.0)	<0.001
April			
Surgery within 2 days	312 (70.1)	452 (75.6)	<0.001
Surgery after 2 days	86 (19.3)	125 (20.9)	·
No surgery	47 (10.6)	21 (3.5)	·
Length of stay, d	10.9 ± 6.0	12.6 ± 6.3	<0.001
Rehabilitation within 30 days	165 (38.3)	371 (63.5)	<0.001
May			
Surgery within 2 days	371 (67.3)	442 (74.8)	0.002
Surgery after 2 days	131 (23.8)	123 (20.8)	·
No surgery	49 (8.9)	26 (4.4)	·
Length of stay, d	11.3 ± 6.5	12.3 ± 8.1	0.027
Rehabilitation within 30 days	258 (48.3)	364 (62.5)	<0.001

* Obtained with chi-squared test or t-test, where appropriate.

Note: Values are count (percentage) or mean ± standard deviation.

In Table 2, we also present the proportion of HF patients that received rehabilitation treatments within 30 days of hospital admission. We observed a significant reduction as compared with 2019, particularly in February (–6.4% [57.9% vs. 64.3%]), March (–20.8% [43.2% vs. 64.0%]), April (–25.2% [38.3% vs. 63.5%]) and May (–14.2% [48.3% vs. 62.5%]).

Table 3 shows the 30-day mortality rates between January and May, and the corresponding adjusted ORs comparing 2020 and 2019. We found a significant increase in mortality in March 2020 (adj. OR = 2.08).

**Table 3:** Thirty-day mortality following hip fracture by year and month of the year, Emilia-Romagna, Italy.

Month of	Year of admission		Odds ratio	*P*-value	Adj.* odds ratio	Adjusted*
admission	2020	2019	95% (CI)		95% (CI)	*P*-value
January	24 (4.1%)	38 (6.1%)	0.66 (0.39–1.11)	0.118	0.67 (0.39–1.15)	0.146
February	28 (5.4%)	24 (4.7%)	1.14 (0.65–2.00)	0.639	1.21 (0.68–2.15)	0.508
March	41 (9.5%)	24 (4.5%)	2.22 (1.32–3.74)	0.003	2.08 (1.23–3.53)	0.007
April	26 (5.8%)	25 (4.2%)	1.42 (0.81–2.50)	0.220	1.48 (0.83–2.64)	0.186
May	33 (6.0%)	24 (4.1%)	1.51 (0.88–2.58)	0.137	1.50 (0.86–2.60)	0.150

*Adjusted by age, sex, and comorbidity index (M-CDS) via logistic regression analysis.

*CI*, confidence interval.

Table 4 summarizes the logistic regression model and shows the association of demographic/clinical characteristics, healthcare process indicators and study period (March 2020 vs. 2019) with 30-day mortality following HF. We found that females had a reduced risk (adj. OR = 0.52), while patients aged ≥90 had an increased risk as compared with those <80 years (adj. OR = 4.33). M-CDS and LOS were not associated with increased 30-day mortality, while undergoing surgery after 48 hours since hospital admission and not receiving surgery were significant risk factors (adj. ORs = 3.08 and 6.19, respectively). Controlling for these factors, HF patients hospitalized in March 2020 were at higher risk of 30-day mortality as compared with those hospitalized in March 2019 (OR = 2.21).

**Table 4:** Association of demographic/clinical characteristics, process indicators and study period (pre-/post-pandemic) with 30-day mortality following hip fracture among patients admitted to the hospital in March, Emilia-Romagna, Italy.

Characteristic	Odds	95% CI	P-value
	ratio		
Sex			
Male	Ref.		
Female	0.52	0.30–0.91	0.023
Age, y			
<80	Ref.		
80–89	2.36	0.95–5.86	0.066
≥90	4.33	1.70–11.04	0.002
M-CDS			
0–1	Ref.		
2–5	0.78	0.30–2.04	0.614
6–9	1.55	0.62–3.89	0.346
≥10	1.29	0.50–3.32	0.597
Surgery			
Within 2 days	Ref.		
After 2 days	3.08	1.59–5.97	0.001
No	6.19	2.86–13.38	<0.001
Length of stay, d			
<7	Ref.		
7–14	0.72	0.37–1.38	0.321
>14	0.97	0.45–2.10	0.947
Study period			
Pre-COVID-19 (2019)	Ref.		
Post-COVID-19 (2020)	2.21	1.27–3.86	0.005

*M-CDS, Modified Chronic Disease Score.

## Discussion

In this observational study, we assessed the impact of the first wave of the COVID-19 pandemic on non-COVID-19 patients with HF. Specifically, we investigated whether the health crisis affected the quality of care provided to elderly patients by analyzing delays in surgical interventions, LOS and timely rehabilitation as process indicators, and 30-day mortality as the main health outcome.

### Statement of principal findings

Our study shows that the pandemic negatively affected non-COVID-19 patients with HF. The quality of care has been undermined by the unavoidable services’ reorganization needed to address the emergency. The proportion of patients undergoing surgery and receiving timely treatment decreased, as well as the mean LOS and the timely use of rehabilitation services. Health outcomes suffered as well: patients with HF experienced an increased mortality rate, particularly in March 2020. In the following months, HF mortality returned to approach pre-crisis levels, demonstrating the adaptation and resilience of the healthcare system.

### Interpretation within the context of the wider literature

Our analysis shows that the first wave of the COVID-19 pandemic determined a significant reduction in HF hospitalizations in Emilia-Romagna, one of the most severely hit areas of Italy and Europe, although the patient characteristics did not differ between 2020 and 2019. This finding is consistent with other studies [21–23] and can be explained by the enforcement of a strict national lockdown from March 9 to May 4, 2020. By confining people at home, interrupting mobility and work activities, and reducing road traffic, the frequency of travel- and work-related injuries dropped; this led to an overall reduction in the number of patients accessing emergency departments and hospitals. Fear of hospitalization could also have been responsible for this reduction [24, 25]. Moreover, COVID-19 infections, hospitalizations and fatalities might have averted the overall number of HFs in the elderly population.

The disruption of the healthcare services determined an increase in the percentage of patients with HF that did not undergo surgery and a decline in the proportion of patients undergoing timely surgery within 48 hours of hospital admission, together with a reduction in mean LOS. These changes could be ascribed to the sudden hospital overload experienced during the first months of 2020, which coerced healthcare institutions to enforce prioritization of their services. Many professionals’ skills, such as surgeons’ and anesthesiologists’, were repurposed to attend to COVID-19 patients in dedicated wards. This created service gaps, reducing both the number of physicians and the time dedicated to non-COVID-19 patients [21]. Diminished healthcare capacity was the reason behind the curb of peri- and post-operative care in HF patients, which is shown by the significantly reduced mean LOS. Following health policy-maker’s suggestions, early discharge was recommended to decrease the risk of hospital-acquired infections and to convert non-COVID-19 hospital beds to COVID-19 beds. Other studies described similar gaps in the healthcare services’ capacities and capabilities during the pandemic and reported similar results [22, 25].

Further considering health services’ performance, we found that the number of patients receiving bed-based rehabilitation within 30 days of hospital admission from February to May 2020 was lower compared with the same period of the previous year. The performance of rehabilitative care could have been undermined by the difficulty to reorganize treatment pathways for non-COVID-19 patients. In Emilia-Romagna, many rehabilitation centers experienced COVID-19 outbreaks, preventing them from providing adequate standards of care and safety [21]. As shown in several studies, the inability of outpatient rehabilitation facilities (i.e., community hospitals and nursing homes) to accept and treat patients coming from acute care hospitals could be responsible for the reduction and delay of timely rehabilitation treatment [26, 27].

Since the initial outbreak of the pandemic, several authors reported an excess of mortality for patients with COVID-19 affected by HF [28–30]. Our study shows that in 2020 the 30-day mortality rate of non-COVID-19 elderly patients with HF was higher compared with the previous year. This increase was significant in March 2020, which was the month with the highest incidence of COVID-19 cases in Emilia-Romagna during our study period (see S1 Fig). The risk of dying (adjusted by age, sex, and comorbidities) was twice as high as the one observed in March 2019. This could be related to the extreme pressure that healthcare structures had to withstand [9, 16], to the abrupt changes in healthcare organization and management, and to the possible lack of attention to the treatment pathways [22, 23, 30].

In keeping with existing literature [28], our analysis confirms that timely surgery after HF was associated with reduced mortality within 30 days of hospital admission. Considering this, it may seem surprising that March 2020 did not exhibit a significant reduction in the proportion of patients undergoing timely surgery, but rather a slight non-significant increase when compared with March 2019. On the one hand, it is possible that in March 2020 there was greater availability of operating rooms and resources to treat HFs due to a reduction in elective surgical procedures for other conditions. One the other hand, the worsening of 30-day mortality in those weeks of 2020 may be marginally explained by a significant reduction in LOS. However, the significant gap in mortality resulting from our multivariable model suggests that other variables should be seen as determinants of patient health after HF, such as misreporting, or misclassification of actual COVID-19 cases and additional factors related to patient clinical management not evaluated in this study.

In the following two months of 2020, 30-day mortality was higher than in April and May 2019, but the gap was not significant and narrower when compared to that observed between March 2019 and March 2020. However, during these months the process indicators remained significantly worse than in 2019. These findings underline the Emilia-Romagna healthcare system’s capacity to respond to the initial health crisis and to take effective actions to mitigate the impact of the pandemic.

### Implications for policy, practice, and research

In this study, we found that the quality of care for patients with HF has been undermined by the unavoidable healthcare services’ reorganization needed to address the COVID-19 pandemic [31]. After the first months of the emergency, HF indicators exhibited a trend to return to pre-crisis levels, suggesting the adaptation and resilience of the healthcare system. Despite the inability to evaluate functional capacities and medium-/long-term healthcare quality indicators, we can assume that the cumulative unmet needs of the patients that did not receive timely surgery and rehabilitation may have led to a worsening of their medium- and long-term outcomes. Considering this, healthcare policymakers and professionals involved in the management of COVID-19 patients should be aware of the needs of patients with other acute and chronic health needs, which should be carefully considered, investigated, and included in future emergency preparedness and response plans.

### Strengths and limitations

Analysis of complete data related to the whole healthcare system of a wide region is the main strength of this study. Moreover, the Italian experience during the first wave of the COVID-19 pandemic represents an instructive event that has the potential to illustrate a healthcare system’s early response to a severe health crisis. Misreporting and misclassification of COVID-19 cases and deaths is the main limitation of our study and is a common problem highlighted elsewhere in the recent literature [32, 33]. There is the possibility that under-diagnosis of COVID-19 among the patients with HF produced an over-estimation in 30-day mortality rates. However, we relied upon the ICD-9-CM classification system issued by Italy’s Ministry of Health and Regional Authorities for the correct identification of COVID-19 cases and deaths. Other limitations are common to all studies based on administrative data, including lack of accuracy and differences in the coding criteria over time, but it is hard to believe that such potential sources of information bias might have significantly affected our estimates.

## Conclusions

This study addressed the impact of the COVID-19-related healthcare reorganization on healthcare quality and 30-day mortality for non-COVID-19 elderly patients with HF. Our results show a reduction in the proportion of patients undergoing surgery and in the proportion of patients receiving timely surgery and rehabilitation. Mortality increased significantly in March 2020 as compared with March 2019, but differences in patient characteristics and quality of care only partially explained such increase. Further studies are needed to verify additional determinants, in order to identify the strengths and weaknesses of healthcare systems and to develop capacities and capabilities suited to face the upcoming public health challenges.

The care and attention required for patients with COVID-19 should not distract from the needs of patients with other critical acute and chronic conditions, which should be carefully investigated and included in future emergency preparedness and response plans.

## Supporting information

S1 Dataset Supporting data. (XLS)

S1 Fig Incidence and prevalence of COVID-19 cases (×100,000 population) in Emilia-Romagna, Italy, between February 24, 2020, and May 31, 2020. Source: *Dipartimento della protezione civile*.
(DOCX)

## References

[1] Veronese Nicola, Maggi Stefania. Epidemiology and social costs of hip fracture, *Injury*. 2018, 49, 1458–60. doi: 10.1016/j.injury.2018.04.015 29699731

[2] Liem IS, Kammerlander C, Suhm N, Blauth M, Roth T, Gosch M, et al. Identifying a standard set of outcome parameters for the evaluation of orthogeriatric co-management for hip fractures. *Injury*. 2013; 44(11):1403–12. doi: 10.1016/j.injury.2013.06.018 23880377

[3] Beaupre LA, Khong H, Smith C, Kang S, Evens L, Jaiswal PK, et al. The impact of time to surgery after hip fracture on mortality at 30- and 90-days: Does a single benchmark apply to all? *Injury*. 2019; 50(4):950–5. doi: 10.1016/j.injury.2019.03.031 30948037

[4] Roberts KC, Brox WT. AAOS Clinical Practice Guideline: Management of Hip Fractures in the Elderly [published correction appears in J Am Acad Orthop Surg. 2015 Apr;23(4):266]. J Am Acad Orthop Surg. 2015;23(2):138–140. doi: 10.5435/JAAOS-D-14-00433 25624366

[5] Tedesco D, Gibertoni D, Rucci P, Hernandez-Boussard T, Rosa S, Bianciardi L, et al. Impact of rehabilitation on mortality and readmissions after surgery for hip fracture. *BMC Health Serv Res*. 2018;18(1):701. doi: 10.1186/s12913-018-3523-x 30200950 PMC6131904

[6] Lavikainen P, Koponen M, Taipale H, Tanskanen A, Tiihonen J, Hartikainen S, et al. Length of Hospital Stay for Hip Fracture and 30-Day Mortality in People With Alzheimer’s Disease: A Cohort Study in Finland. *J Gerontol A Biol Sci Med Sci*. 2020; 75(11):2184–92. doi: 10.1093/gerona/glaa199 32797165 PMC7566552

[7] Moyet J, Deschasse G, Marquant B, Mertl P, Bloch F. Which is the optimal orthogeriatric care model to prevent mortality of elderly subjects post hip fractures? A systematic review and meta-analysis based on current clinical practice. *Int Orthop*. 2019; 43(6):1449–54. doi: 10.1007/s00264-018-3928-5 29691612

[8] Brugel M, Carlier C, Essner C, Debreuve-Theresette A, Beck MF, Merrouche Y, et al. Dramatic Changes in Oncology Care Pathways During the COVID-19 Pandemic: The French ONCOCARE-COV Study. *Oncologist*. 2021; 26(2):e338–e341. doi: 10.1002/onco.13578 33111460 PMC7873337

[9] Richards M., Anderson M., Carter P., Ebert B. L., Mossialos E. The impact of the COVID-19 pandemic on cancer care. *Nat Cancer* 1, 565–7 (2020). 10.1038/s43018-020-0074-y PMC7238956 35121972

[10] Randelli P.S., Compagnoni R. Management of orthopaedic and traumatology patients during the Coronavirus disease (COVID-19) pandemic in northern Italy. *Knee Surg Sports Traumatol Arthrosc* 28, 2020. 1683–9. 10.1007/s00167-020-06023-3 PMC7183254 32335697

[11] Hadfield J. N., & Gray A. C. The Evolving COVID-19 Effect on Hip Fracture Patients. *Injury*, 2020, 51(7), 1411–2. doi: 10.1016/j.injury.2020.06.006 32553412 PMC7295511

[12] Di Martino A., Faldini C. Trauma service reorganization in Bologna (Italy) during COVID-19 pandemic. *Injury*, 2020, 51(7), 1684. doi: 10.1016/j.injury.2020.04.033 32386838 PMC7187857

[13] Liebensteiner M. C., Khosravi I., Hirschmann M. T., Heuberer P. R., Board of the AGA-Society of Arthroscopy and Joint-Surgery, Thaler M. Massive cutback in orthopaedic healthcare services due to the COVID-19 pandemic. *Knee surgery*, *sports traumatology*, *arthroscopy*: *official journal of the ESSKA*, 2020, 28(6), 1705–11. 10.1007/s00167-020-06032-2 PMC7192059 32356047

[14] Slullitel PA, Lucero CM, Soruco ML, Barla JD, Benchimol JA, Boietti BR, et al. Prolonged social lockdown during COVID-19 pandemic and hip fracture epidemiology. *Int Orthop*. 2020; 44(10):1887–95. doi: 10.1007/s00264-020-04769-6 32772318 PMC7414899

[15] Napoli N, Elderkin AL, Kiel DP, Khosla S. Managing fragility fractures during the COVID-19 pandemic. *Nat Rev Endocrinol*. 2020 Sep;16(9):467–8. doi: 10.1038/s41574-020-0379-z 32528045 PMC7288256

[16] Golinelli D, Bucci A, Adja KYC, Toscano F. Comment on: "The Italian NHS: What Lessons to Draw from COVID-19?". *Appl Health Econ Health Policy*. 2020;18(5):739–41. doi: 10.1007/s40258-020-00608-2 32833141 PMC7443390

[17] Caminiti C, Maglietta G, Meschi T, Ticinesi A, Silva M, Sverzellati N. Effects of the COVID-19 Epidemic on Hospital Admissions for Non-Communicable Diseases in a Large Italian University-Hospital: A Descriptive Case-Series Study. *Journal of Clinical Medicine*. 2021; 10(4):880. doi: 10.3390/jcm10040880 33669906 PMC7924591

[18] Donabedian A. The quality of care. How can it be assessed? *JAMA*. 1988; 260(12):1743–8. doi: 10.1001/jama.260.12.1743 3045356

[19] Golinelli D, Boetto E, Mazzotti A, Rosa S, Rucci P, Berti E, et al. Cost Determinants of Continuum-Care Episodes for Hip Fracture. *Health Serv Insights*. 2021; 14:1178632921991122. doi: 10.1177/1178632921991122 33642863 PMC7894600

[20] Iommi M, Rosa S, Fusaroli M, Rucci P, Fantini MP, Poluzzi E. Modified-Chronic Disease Score (M-CDS): Predicting the individual risk of death using drug prescriptions. *PLoS One*. 2020; 15(10):e0240899. doi: 10.1371/journal.pone.0240899 33064757 PMC7567358

[21] Persiani P, De Meo D, Giannini E, Calogero V, Speziale Varsamis T, Cavallo AU, et al. The Aftermath of COVID-19 Lockdown on Daily Life Activities in Orthopaedic Patients. *J Pain Res*. 2021; 14:575–83. doi: 10.2147/JPR.S285814 33688247 PMC7936667

[22] Santi L, Golinelli D, Tampieri A, Farina G, Greco M, Rosa S, et al. Non-COVID-19 patients in times of pandemic: Emergency department visits, hospitalizations and cause-specific mortality in Northern Italy. *PLoS One*. 2021; 16(3):e0248995. doi: 10.1371/journal.pone.0248995 33750990 PMC7984614

[23] Golinelli D, Campinoti F, Sanmarchi F, et al. Patterns of Emergency Department visits for acute and chronic diseases during the two pandemic waves in Italy [published online ahead of print, 2021 Jul 9]. Am J Emerg Med. 2021;50:22–26. doi: 10.1016/j.ajem.2021.07.010 34271231 PMC9758955

[24] Baldi E, Savastano S. Fear of Contagion: One of the Most Devious Enemies to Fight During the COVID-19 Pandemic. *Disaster Med Public Health Prep*. 2020; 1–2. doi: 10.1017/dmp.2020.338 32907670 PMC7653481

[25] Wheaton MG, Prikhidko A, Messner GR. Is Fear of COVID-19 Contagious? The Effects of Emotion Contagion and Social Media Use on Anxiety in Response to the Coronavirus Pandemic. *Front Psychol*. 2021; 11:567379. doi: 10.3389/fpsyg.2020.567379 33469434 PMC7813994

[26] Istituto Superiore Di Sanità: Donfrancesco C., Lo Noce C., Bacigalupo I., D’Ancona P.F., Galati F., Di Lonardo A., et al. SORVEGLIANZA STRUTTURE RESIDENZIALI SOCIO-SANITARIE NELL’EMERGENZA COVID-19: Report Nazionale Andamento temporale dell’epidemia di COVID-19. *ISS*. 2021. available at: https://www.epicentro.iss.it/coronavirus/sars-cov-2-sorveglianza-rsa

[27] American Geriatrics Society. American Geriatrics Society Policy Brief: COVID-19 and Nursing Homes. *J Am Geriatr Soc*. 2020; 68(5):908–11. doi: 10.1111/jgs.16477 32267538 PMC7262210

[28] Rasidovic D, Ahmed I, Thomas C, Kimani PK, Wall P, Mangat K, NOF-COV19 Study Collaborative Group. Impact of COVID-19 on clinical outcomes for patients with fractured hip: a multicentre observational cohort study. *Bone Jt Open*. 2020; 1(11):697–705. doi: 10.1302/2633-1462.111.BJO-2020-0132.R1 33263109 PMC7690757

[29] Clement ND. Letter to the editor: Coronavirus disease 2019 (COVID-19) markedly increased mortality in patients with hip fracture: A systematic review and meta-analysis. *J Clin Orthop Trauma*. 2021; 12(1):43. doi: 10.1016/j.jcot.2020.11.007 33239854 PMC7673211

[30] Campo G., Fortuna D., Berti E., De Palma R., Di Pasquale G., Galvani M., et al. In- and out-of-hospital mortality for myocardial infarction during the first wave of the COVID-19 pandemic in Emilia-Romagna, Italy: A population-based observational study. *The Lancet Regional Health Europe*, 2021, 0-0-100055. 10.1016/j.lanepe.2021.100055 PMC8454529 34557800

[31] Golinelli D, Lenzi J, Adorno E, Gianino MM, Fantini MP. COVID-19 and regional differences in the timeliness of hip-fracture surgery: an interrupted time-series analysis. PeerJ. 2021;9:e12046. Published 2021 Aug 31. doi: 10.7717/peerj.12046 34540366 PMC8415287

[32] Sanmarchi F, Golinelli D, Lenzi J, et al. Exploring the Gap Between Excess Mortality and COVID-19 Deaths in 67 Countries. JAMA Netw Open. 2021;4(7):e2117359. doi: 10.1001/jamanetworkopen.2021.17359 34269809 PMC8285734

[33] Gibertoni D, Sanmarchi F, Adja KYC, et al. Small-scale spatial distribution of COVID-19-related excess mortality. MethodsX. 2021;8:101257. doi: 10.1016/j.mex.2021.101257 33996519 PMC8105045

