# Supplementary material for: Variations of the quality of care during the COVID-19 pandemic affected the mortality rate of non-COVID-19 patients with hip fracture
Source: PLoS One. 2022 Feb 16;17(2):e0263944. doi: 10.1371/journal.pone.0263944 (PMC8849602; doi:10.1371/journal.pone.0263944)

**S1 Fig. Incidence and prevalence of COVID-19 cases (×100,000 population) in Emilia-Romagna, Italy, between February 24, 2020, and May 31, 2020.** Source: *Dipartimento della protezione civile*.


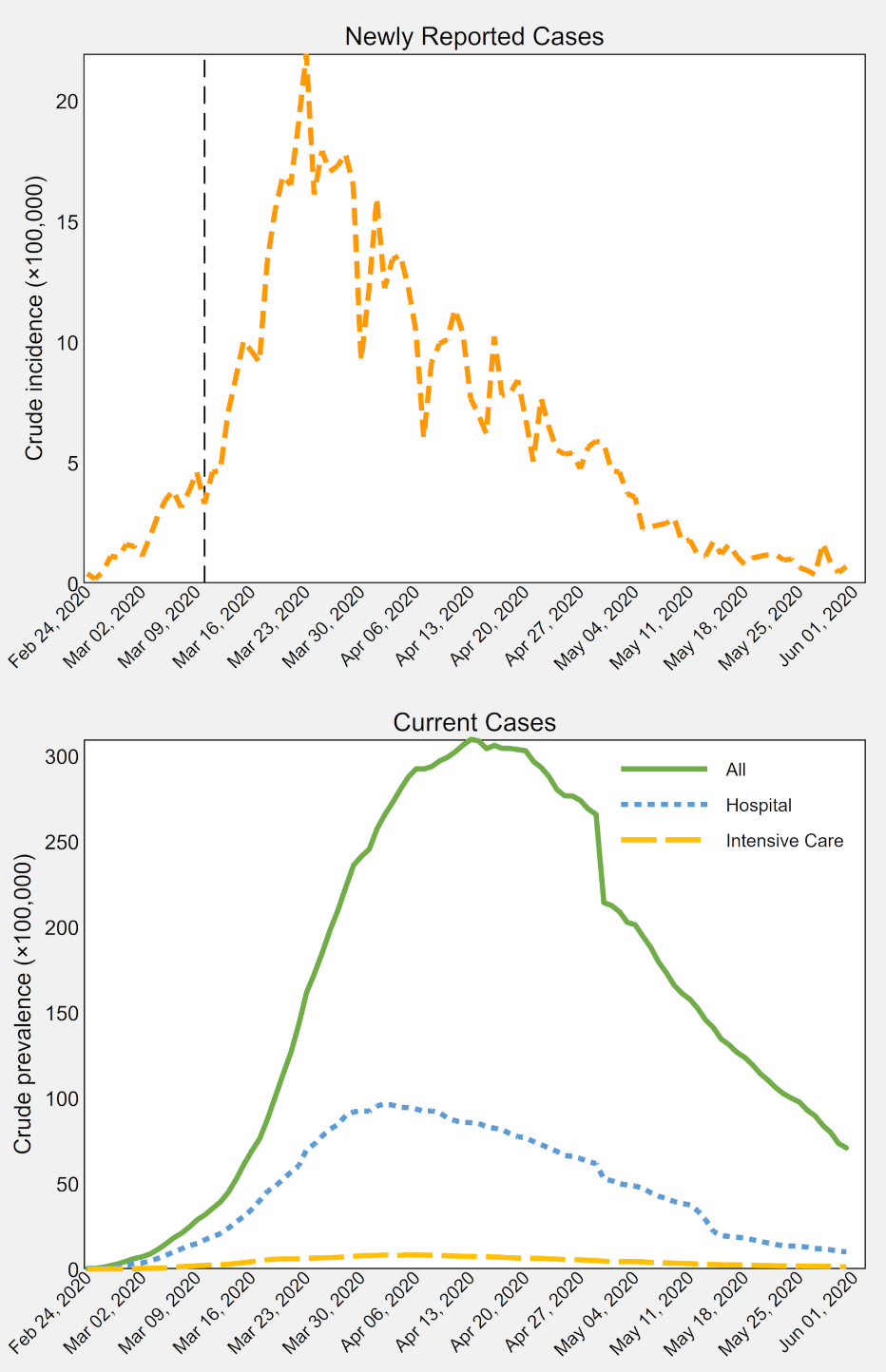

Supplement: S1 Fig — Source: Dipartimento della protezione civile. (DOCX) [file pone.0263944.s002.docx]
